# A Novel Alkaliphilic Bacillus Esterase Belongs to the 13^th^ Bacterial Lipolytic Enzyme Family

**DOI:** 10.1371/journal.pone.0060645

**Published:** 2013-04-05

**Authors:** Lang Rao, Yanfen Xue, Yingying Zheng, Jian R. Lu, Yanhe Ma

**Affiliations:** 1 State Key Laboratory of Microbial Resources, Institute of Microbiology, Chinese Academy of Sciences, Beijing, China; 2 The Graduate School, Chinese Academy of Sciences, Beijing, China; 3 Biological Physics Laboratory, School of Physics and Astronomy, the University of Manchester, Manchester, United Kingdom; Massachusetts Institute of Technology, United States of America

## Abstract

**Background:**

Microbial derived lipolytic hydrolysts are an important class of biocatalysts because of their huge abundance and ability to display bioactivities under extreme conditions. In spite of recent advances, our understanding of these enzymes remains rudimentary. The aim of our research is to advance our understanding by seeking for more unusual lipid hydrolysts and revealing their molecular structure and bioactivities.

**Methodology/Principal Findings:**

Bacillus. *pseudofirmus* OF4 is an extreme alkaliphile with tolerance of pH up to 11. In this work we successfully undertook a heterologous expression of a gene *estof4* from the alkaliphilic *B. pseudofirmus* sp OF4. The recombinant protein called EstOF4 was purified into a homologous product by Ni-NTA affinity and gel filtration. The purified EstOF4 was active as dimer with the molecular weight of 64 KDa. It hydrolyzed a wide range of substrates including p-nitrophenyl esters (C2–C12) and triglycerides (C2–C6). Its optimal performance occurred at pH 8.5 and 50°C towards p-nitrophenyl caproate and triacetin. Sequence alignment revealed that EstOF4 shared 71% identity to esterase Est30 from *Geobacillus stearothermophilus* with a typical lipase pentapeptide motif G91LS93LG95. A structural model developed from homology modeling revealed that EstOF4 possessed a typical esterase 6α/7β hydrolase fold and a cap domain. Site-directed mutagenesis and inhibition studies confirmed the putative catalytic triad Ser93, Asp190 and His220.

**Conclusion:**

EstOF4 is a new bacterial esterase with a preference to short chain ester substrates. With a high sequence identity towards esterase Est30 and several others, EstOF4 was classified into the same bacterial lipolytic family, Family XIII. All the members in this family originate from the same bacterial genus, bacillus and display optimal activities from neutral pH to alkaline conditions with short and middle chain length substrates. However, with roughly 70% sequence identity, these enzymes showed hugely different thermal stabilities, indicating their diverse thermal adaptations via just changing a few amino acid residues.

## Introduction

Enzymes are essential molecules that command thousands of reactions in living systems. They control reaction rates and specificities and regulate multiple reaction processes under complex chemical and biological environments. Distinctly different from man-made catalysts, they enable these catalytic processes to occur under bodily temperatures and are thus highly energy efficient. Moreover, enzymes can also sustain life under extreme pH and salt whilst still retaining remarkable efficiencies and stability.

Extensive research has been undertaken to understand how enzymes work and encompass these unique features because advances can lead to better healthcare and technological exploitation. Enzymes are already of widespread use in food and beverage, textiles and papers, with fast uptake in chemical and pharmaceutical sectors over the past few decades. Esterases (EC 3.1.1.1) and lipases (EC 3.1.1.3) are lipolytic enzymes widely used for the hydrolysis of ester bonds and transesterification. Even though their substrate specificities differ, they share similar catalytic mechanisms and molecular structures. These lipolytic reactions are inherently associated with the α/β hydrolase superfamily and share the same catalytic process via the Ser-Asp-His (or Glu to replace Asp) triad and the same molecular structure of the α/β hydrolase folds [1], [2]. Because of their different substrate specificities, stability and region-stereo specificities, these enzymes are attractive to a wide range of industrial applications including food and drink, fine chemicals and pharmaceuticals [3], [4].

Microorganisms live in vastly different natural environments, ranging from salty and alkali wet lands, cold deep sea beds to hot springs. Those selected from extreme environments have become the attractive resources for exploring novel enzymes with unusual stability against thermal, alkaline and organic solvent conditions [5], [6]. Prokaryotes-derived lipolytic enzymes were first categorized into 8 families by Arpigny and Jeager [7]. The division was made on the base of sequence identity and biochemical properties and proved to be useful against the fast increasing number of bacterial lipases and esterases. The true lipase family, Family I, covered the 6 subfamilies which principally catalyzed the hydrolytic reactions on substrates with long acyl chains. Carboxyl esterase families were grouped into Family II (also called GDSL family), Family III, Family IV (also called HSL family), Family V, Family VI, Family VII and Family VIII [7]. Subsequent studies led to the discovery of new enzymes that could not be grouped in the existing 8 families. The 9^th^ family of bacterial esterases (Family IX) was created when nPHB depolymerase PhaZ7 was discovered [8]. The hyperthermophilic esterase EstD from *Thermotoga maritime* was categorized into Family X. Metagenomic derived lipolytic enzymes LipG [9] and LipEH166 [10] were the 11^th^ and 12^th^ bacterial esterase family members. Family XIII started from the discovery of esterase Est30 from *Geobacillus stearothermophilus* [11], [12] through 3D structural characterisation that revealed a three-helix cap domain and an α/β hydrolase fold domain with a peculiar topological structure. The most recently found bacterial lipolytic family is Family XIV with thermostable esterase EstA3 as typical member [13].

In this work we report the discovery of a novel esterase EstOF4 from *Bacillus pseudofirmus* OF4. This enzyme showed high sequence identity to the bacterial esterase Est30 in Family XIII. In order to elucidate the phylogenic status of esterase EstOF4 and explore its catalytic potential, we have analyzed the primary sequence and 3D structure of this enzyme and assessed the enzymatic properties. Comparison of EstOF4 against all the members from Family XIII was also undertaken and the key features for this bacterial esterase family were discussed.

## Materials and Methods

### Bacterial strains, plasmids, enzymes and chemicals

pET-28a (Novagen, Germany) was used as expression vector. *E. coli* DH5α and *E. coli* BL21(DE3) (Novagen, Germany) used as host strains for gene duplication and protein expression, respectively. Luria-Bertani (LB) medium was used for bacterial culture with 50 µg/ml kanamycin as antibiotics and IPTG (0.5 mM) was added in the medium to induce protein expression. *B. pseudofirmus* OF4 was a gift from Dr Terry and was cultured in malate-containing medium as described [14]. Restriction enzymes *Nde*I and *BamH*I were purchased from Promega. Ultrapure deoxynucleotide solution (dNTPs) was purchased from Pharmacia Biotech (Sweden). Ex-Taq DNA polymerase, T4DNA ligase and DNA Marker were purchased from TaKaRa Biotechnology (Dalian, China). Isopropylthio-β-D-galactoside (IPTG), *p*-nitrophenyl acetate (*p*NPC2), *p*-nitrophenyl butyrate (*p*NPC4), *p*-nitrophenyl caproate (*p*NPC6), *p*-nitrophenyl caprylate (*p*NPC8), *p*-nitrophenyl caprate (*p*NPC10), *p*-nitrophenyl laurate (*p*NPC12), *p*-nitrophenyl palmitate (*p*NPC16), β-naphthyl acetate, Fast blue and gel filtration molecular weight markers were purchased from Sigma (Sigma, USA). The IEF marker was purchased from Serva electrophoresis (Heidelberg, Germany). All other chemicals were of the highest reagent grade commercially available.

### Cloning of esterase gene and construction of the expression vector

The esterase gene *estof4* from *B. pseudofirmus* OF4 was amplified by PCR using sense primer *estof4*-up GTGACATATGAAAGTAGTAGCACCTAAGCC, (the *Nde*I site was underlined) and anti-sense primer *estof4*-down GGACGGATCCACAGTCCAGTCTAATCCTTC (the *BamH*I site was underlined). The PCR products were double digested with *Nde*I/*BamH*I and then ligated into *Nde*I/*BamH*I digested plasmid pET-28a. The resultant recombinant plasmid was designated as pET-*estof4* and transformed into *E. coli* DH5α for DNA sequencing. The nucleotide sequence of the recombinant *estof4* was deposited in Genbank with accession number KC579466.

### Expression and purification of esterase EstOF4

Recombinant plasmid pET-*estof4* was transformed into *E. coli* BL21 (DE3) and the recombinant cells were inoculated into 200 ml LB media at 37 °C. When the cell density at OD600 reached 0.8, IPTG (0.5 mM) was added in the media to induce protein expression. Induced cells were harvested by centrifugation at 5,000×g for 10 min and resuspended in 50 mM Tris–HCl buffer (pH 8.0). After ultrasonic cell disintegration, the cell debris was removed by centrifugation (12000 rpm, 20 min, and 4 °C). The supernatant was applied to a Nickel affinity column (Ni-His NTA Novagen). After desalting, the sample was passed through a Superdex200 10/30 gel filtration column (Amersham Pharmacia Biotech) in 50 mM Tris-HCl (pH 8.0) containing 0.1 M NaCl for further purification. The fractions showing esterase activity were collected and concentrated as purified enzyme.

### Molecular determination, SDS-PAGE and activity staining

The molecular weight of native EstOF4 was determined by gel filtration chromatography using Superdex200 10/30 column. Cytochrome C (12.4 kDa), carbonic anhydrase (29 kDa), bovine serum albumin (66 kDa) and alcohol dehydrogenase (150 kDa) were used as molecular mass standards. Dextran blue (2000 KD) was used to determine the void volume.

The protein concentration was determined using Bio-Rad Protein Assay Kit (Hercules, CA, USA) with bovine serum albumin as the standard. SDS-PAGE was performed according to the description by Laemmli [15]. Esterase activity staining (Zymogram) was performed after non-denaturing PAGE (12% T, 5% C) by incubation of the gel in a solution of 50 mg α-naphthyl acetate and 30 mg Fast Blue RR Salt in 100 ml of 25 mM Tris/HCl, pH 8, 1% (v/v) acetone.

### Enzyme assay, acyl chain length preference and kinetic measurements

Enzyme activity was monitored spectrophotometrically at 410 nm by a DU800 spectrophotometer (Beckman) with *p*-nitrophenyl acylates as substrates. One unit of enzyme activity was defined as the amount of enzyme that releases 1 µmol *p*-nitrophenol from *p*-nitrophenol ester per min. The kinetic parameters *K*_m_ and *k*_cat_ were calculated in 50 mM Tris-HCl buffer (pH 8.5) at 50°C with different *p*NP esters. Kinetic analysis by curve fitting (Hyperbola) was undertaken with GraphPad Prism 5.0 (GraphPad Software, Inc). Activity was also determined by pH end-point titration with triglycerides as substrates. The reactions were done in a glass vessel thermostated at 50 °C containing 15 mL of 50 mM Tris–HCl, 1% substrate (glyceryl esters) in 1% emulsifier (Gum acacia) and 1 mg of esterase. The released free fatty acids were titrated with 0.05 M NaOH up to pH 10.0 using a TitroLine Easy (Schott) titrator. Again, one unit (U) of enzymatic activity was defined as the release of 1 µmol of fatty acid per min.

### Enzymatic property characterization

The effect of pH on enzymatic activity was determined spectrophotometrically using *p*NPC6 as substrate as described above. The substrate was prepared in 50 mM buffer under different pH values: sodium phosphate (pH 7.0 to 8.0), Tris-HCl (pH 8.0 to 9.0), glycine-NaOH (pH 9.0 to 10.5) and Na_2_HPO_4_/NaOH (pH 11.0 to 11.5), with ionic strengths kept constant. The optimum temperature for esterase activity was determined over a range of 20–80°C using DU800 spectrophotometer (Beckman) connected with a temperature control module. The effect of metal ions to the activity of esterase was determined by adding various metal ions (final concentrations at 1 mM) to enzymatic assay solution. The activity of EstOF4 in buffers without adding any ions was taken as 100%.The effect of inhibitors on esterase activity was determined using solutions containing 1 mM EDTA, PMSF and DTT. The effects of organic compounds were determined by adding different organic solvents into reaction solution.

### Site-directed mutagenesis

The East Mutagenesis System kit (TransGen, Beijing) was used to introduce amino acid substitutions, with all manipulations made following the standard protocol. The pET-*estof4* expression plasmid was used as mutation template. Nucleoside base substitutions were introduced by mutagenic primer pairs (introduced alternated nucleoside bases were shown in lowercase letters):

Ser93Ala F 5′AATTGCGGTGTGCGGGCTTgCTCTTGGCGG3′

Ser93Ala R 5′cAAGCCCGCACACCGCAATTTCCTCATGACC3′

Asp190Asn F 5′GTGGTTCAGGCTAGGAATaATGAGATGATTGAC3′

Asp190Asn R 5′tTATTCCTAGCCTGAACCACAAAAGTTGGAGA3′

His220Leu F 5′ GTGGTTCAGGCTAGGAATaATGAGATGATTGAC 3′

His220Leu R 5′aGGCCTGATTCCTCATACCATTTTAATGATTTTTC3′.

The PCR reaction products were purified by agarose gel purification kit (Omega, USA). After digested with DpnI the mutated plasmids were examined by DNA sequencing (Sangon, China). The three mutant *estof4* DNA sequences as shown above have been deposited into GenBank with the accession numbers as KC579467 (for Ser93Ala), KC579468 (Asp190Asn) and KC579469 (His220Leu), respectively.

### Determination of the isoelectric point of the EstOF4

Isoelectric focusing (IEF) of the purified EstOF4 was carried out with precast IEF gel using an Ettan IPGphor 3 Isoelectric Focusing System (GE Healthcare). The IEF was performed in five steps (200 V for 1 h, 500 V for 1 h, 1000 V for 1 h, followed by a gradient switch of 1000–4000 V for 1.0 h and a final 4000 V continuation until the total volt-hours is reached 40 k·Vh) at 20°C. Proteins were visualized by Coomassie Brillliant Blue.

### Sequence analysis and homology modeling

Protein sequence similarity searches were undertaken using BLASTP [16]. Multiple sequence alignments were performed with the Clustal X[17] with default parameters and the alignment figure was exported by ESPript [18]. N-terminal sequence analysis was performed with SignalP3.0 Server [19]. The phylogenetic tree was built using the MEGA4.0 software with the neighbor-joining method [20].

Esterase Est30 from Geobacillus Stearothermophilus (GenBank Number AY186197,Protein Data Bank code 1TQH) shared 71% identity of the amino acid sequence with EstOF4. It was chosen as the best structure template for the homology modeling of the EstOF4. Following alignment, 10 modeled structures of EstOF4 were generated with the MODELLER (version 9.9) program using default parameters [21]. The best one was picked out for following discussion according to the energy function score and the quality of the optimized model was verified by PROCHECK [22]. All 3D structures of EstOF4 proteins were visualized by Pymol Molecular Graphics System [23].

## Results

### Analysis of the sequence of EstOF4

**Figure 1:**
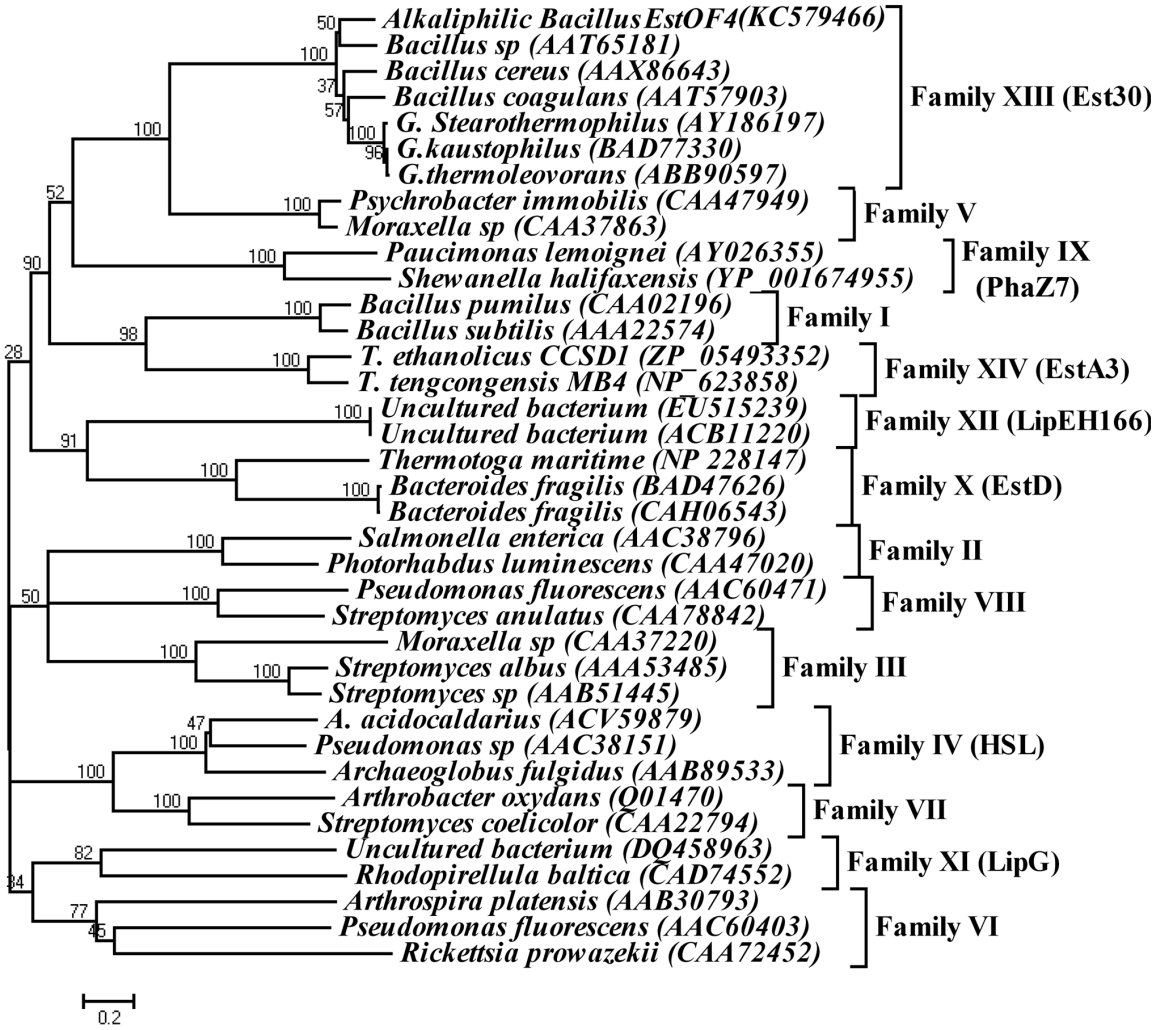
Unrooted neighbor-joining phylogenetic tree of EstOF4 with respect to the relevant lipolytic enzymes and respective families. The amino acid sequences of the lipolyitc enzymes were referred to the previously classified 8 lipolytic families and the newly explored families:Family IX (LipG), Family X (EstD), Family XI (PhaZ7), Family XII (LipEH166) and Family XIII (Est30). Sequence alignment was performed by Clustal W version 1.81 and the tree was created by program MEGA version 4.1.

**Figure 2:**
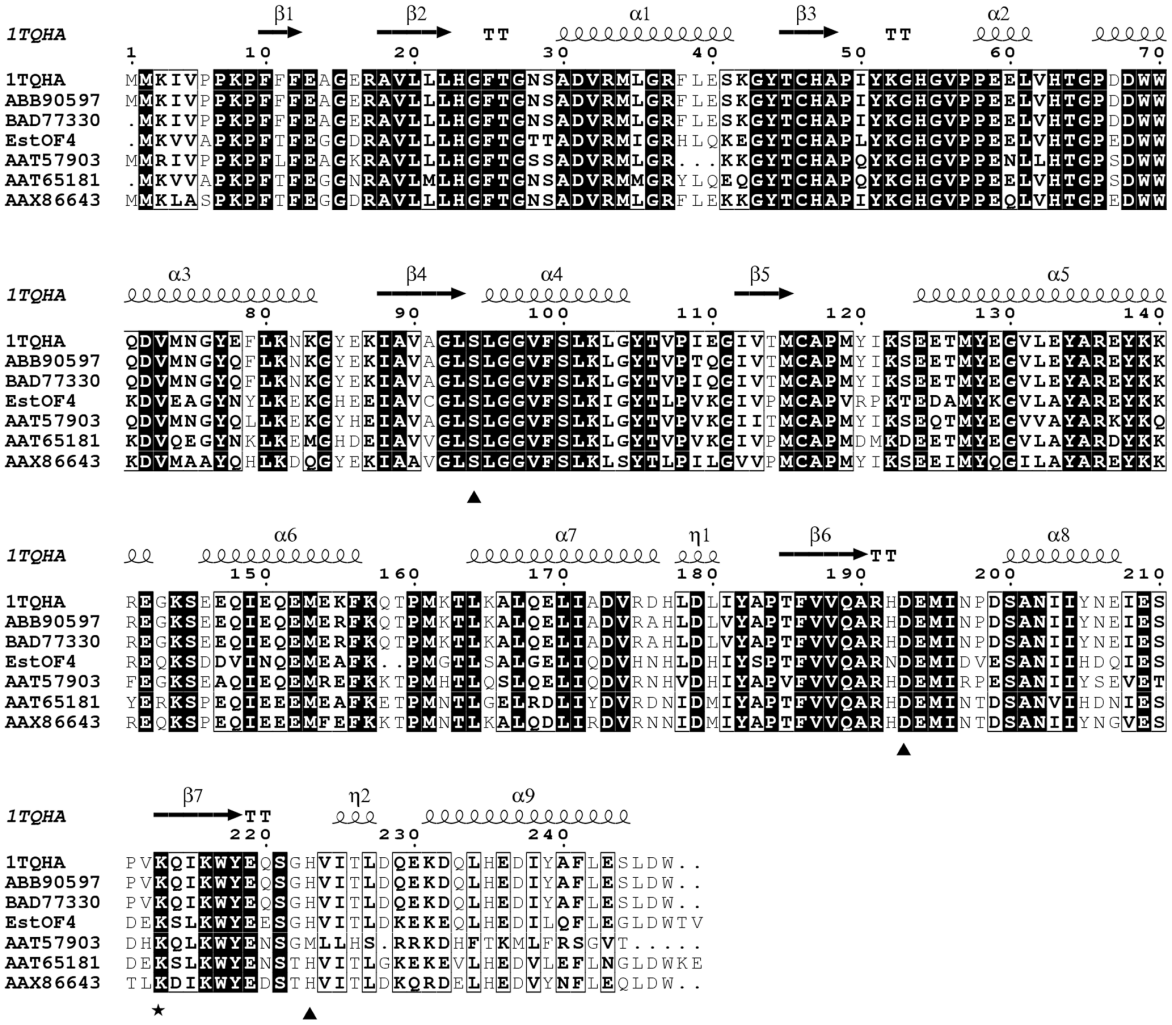
Alignment of EstOF4 sequence with those from previously characterized homologous esterases [AAX86643, AAT57903, AY186197, BAD77330, AAT65181 and ABB90597]. Strictly conserved residues are white letters on grey background, and conservatively substituted residues are boxed. The residues equivalent to the putative acid/base catalysts are indicated by a solid triangle and proteolytic cut site K210 is indicated by black solid circle. The secondary structure elements (helices α, strands β, and helices (η) of 1TQHA (AY186197) are shown above the alignment. The figure was produced using ESPript.

With the genomic information of *B. pseudofirmus OF4*, an open reading frame with 741 bp encoding a polypeptide with 246 amino acids showing striking similarity towards esterase/lipase family proteins was found. Here we designated the protein as EstOF4. BlastP search of the non-redundant analysis of the amino acid sequence of EstOF4 showed that the protein was highly similar to carboxylesterases from *Bacillus species*. Six esterases with enzymatic properties share the highest similarity to EstOF4: AAT65181 (*Bacillus sp*. 01–855 with identity of 75%) [24], AY186197 (*Geobacillus Stearothermophilus*, 71%) [11], [12], ABB90597 (*Geobacillus thermoleovorans*, 71%) [25], BAD77330 (*Geobacillus kaustophilus*, 71%) [26], AAX86643 (*Bacillus cereus* C71, 69%) [27] and AAT57903 (*Bacillus coagulans* 81–11, 68%) [28]. In order to identify the precise relationship between EstOF4 and other known lipolytic proteins, a phylogenetic tree was constructed (Figure 1), based on the 37 sequences representing the hitherto identified fourteen bacterial lipolytic hydrolysis families. Phylogenetic study clearly showed that the sequence of EstOF4 was most close to those in the newly defined esterase Est30 [11]. Figure 2 shows sequence alignments of EstOF4 with esterases from Family Est30. The results indicate that EstOF4 shares the typical serine catalysis pentapeptide GLSLG in protein sequence from 90 to 94. Amino acids Ser93, Asp190 and His220 were also predicted as the catalytic triad of the enzyme since they were highly conserved with esterase Est30.

### Expression and purification of recombinant EstOF4

**Figure 3:**
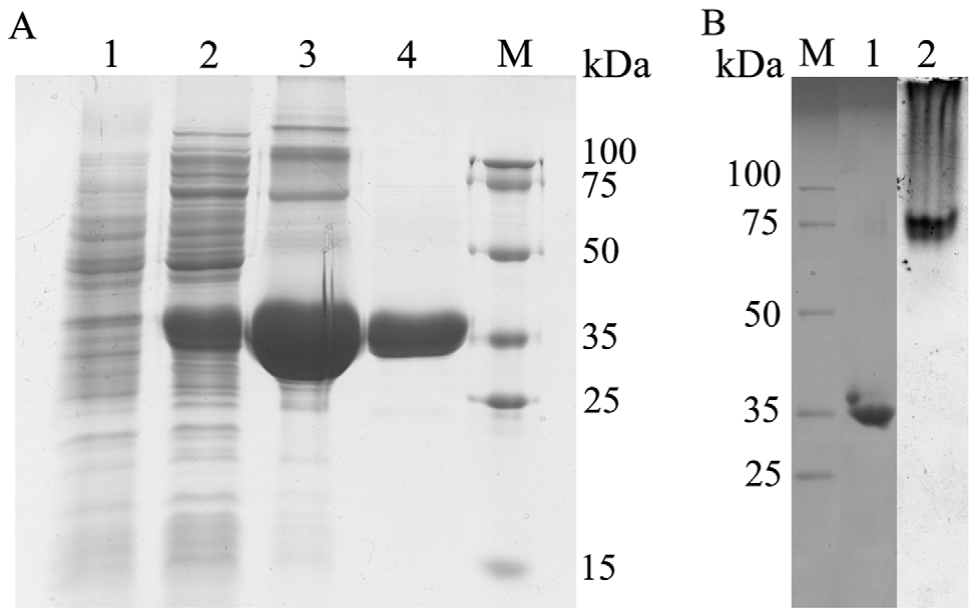
SDS-PAGE analysis of the purification process of esterase EstOF4 and active staining. A: Lane 1: crude supernatant of E. coli BL21; Lanes 2: Lysate of induced E. coli BL21 pET-estof4 cells; Lane 3: purification on Ni-NTA affinity column; Lane 4: further purification with Sephadex-G200; Lane M: molecular weight markers. B: Line 1: denaturing gel electrophoresis of protein EstOF4 stained with Coomassie Blue. Line 2: native gel electrophoresis of EstOF4, the gel was stained with a-naphthyl acetate and Fast Blue for detection of hydrolase activity.

**Figure 4:**
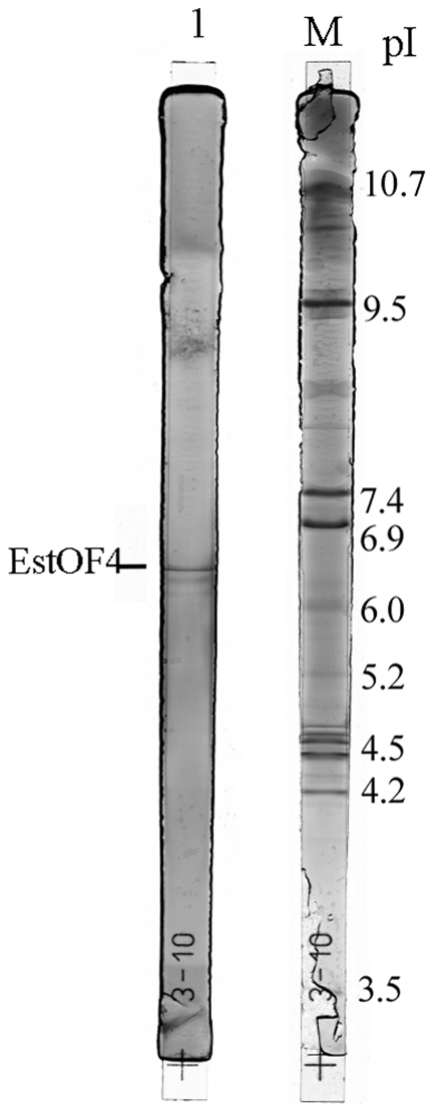
The protein isoelectric point of esterase EstOF4 was determined by isoelectric focusing on precast IEF gel. Lane1: purified esterase EstOF4. Lane M: protein IEF marker contains proteins with pI 3.5 to 10.7.

No signal peptide was detected from the analysis by SignalP. The full sequence of *estof4* was amplified and cloned into expression vector pET28a. The recombinant plasmid pET-*estof4* was then transformed into *E. coli* BL21(DE3) for protein expression. The expression was optimized by induction with various concentrations of isopropylthio-β-D-galactoside (IPTG) under different temperatures. The highest amount of active protein production was achieved when *E. coli* BL21(DE3) was induced overnight with 0.5 M IPTG at 25°C. The heterologously expressed protein EstOF4 with an N-terminal His tag was purified to homogeneity by Ni-NTA affinity and gel filtration chromatography (Superdex200 HR10/30 column), with the final yield of 20% (Table S1 in File S1). SDS-PAGE analysis of the purified protein showed that under denaturing condition its molecular weight (MW) was around 35 KDa (Figure 3). Size exclusion chromatography revealed that in native form the enzyme had a MW of 64 KDa (Figure S1), indicating that EstOF4 was active in homodimeric form. Zymographic staining on a renatured SDS-PAGE gel didn't show hydrolysis band when staining with 1-naphthylacetate/Fast Blue, just like esterases from *B. subtilis, B. coagulans* [29] and *G. stearothermophilus* [30], but a non-denaturing PAGE revealed active band with apparently bigger molecular mass, which implies that the enzyme might be sensitive to SDS and the active form of enzyme could be a dimer or even bigger multimeric forms (Figure 3B). Isoelectric focusing assay revealed that the purified esterase EstOF4 had an isoelectric point (pI) around 6.5 (Figure 4).

### Effect of pH and temperature

**Figure 5:**
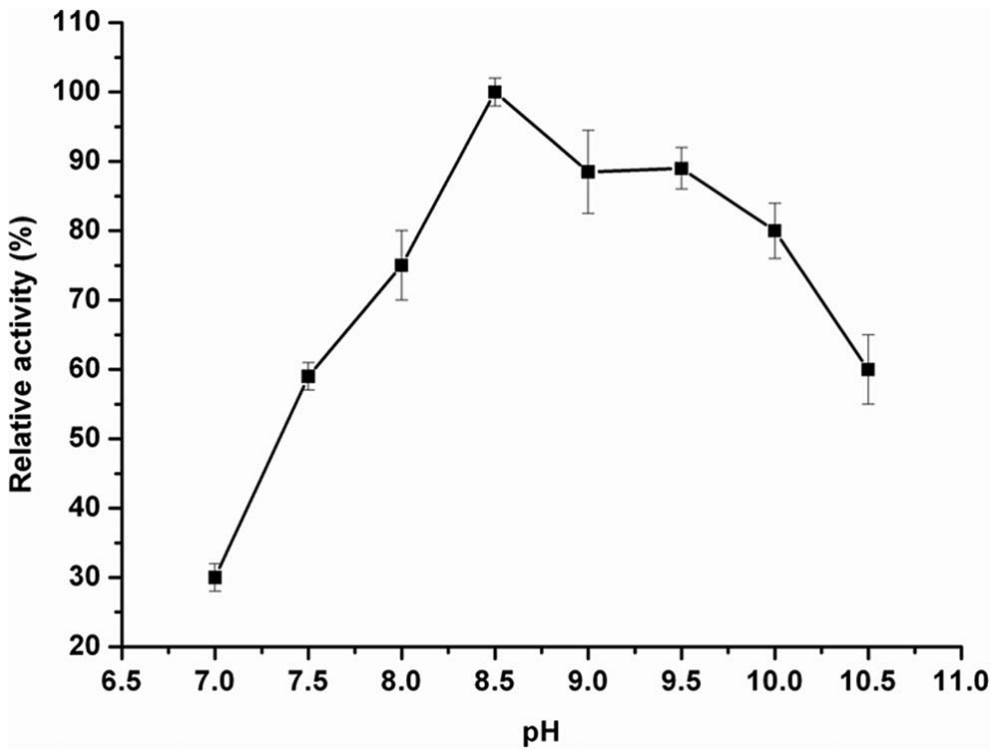
Effect of pH on the activity of esterase EstOF4. The reaction was taken at 50°C with pNC6 as substrate in different pH solution: sodium phosphate (pH 7.0 to 8.0), Tris-HCl (pH 8.0 to 9.0), glycine-NaOH (pH 9.0 to 10.5) and Na2HPO4/NaOH (pH 11.0 to 11.5). Each value of the assay was the arithmetic mean of triplicate measurements. Where pH overlapping, the averaged values were used.

**Figure 6:**
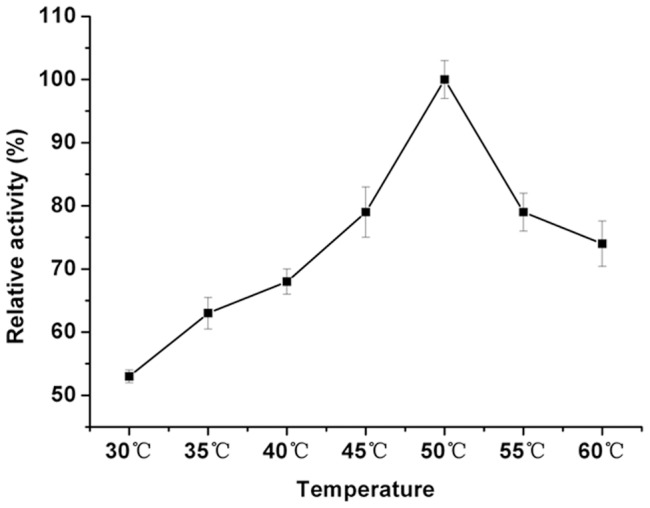
Effect of temperature on the activity of esterase EstOF4. The assay was taken at the optimal pH of 8.5 with pNC6 as substrate. Each value was the averaged over three duplicates.

The effects of pH and temperature on the enzymatic activity of EstOF4 were assayed spectrophotometrically using *p*-nitrophenyl caproate as substrate, with results shown in Figures 5 and 6. The optimal pH of the enzyme was found to be pH 8.5, and the enzyme showed strong activity in alkaline conditions from pH 8–10.5, similar to several other enzymes also from alkaline environment [30], [31], [32]. The enzyme also presented good stability in alkaline condition with 80% residual activity after incubated in pH 10.5 for 12 hours (data not shown). In addition, EstOF4 displayed increasing activity from temperature 30°C to 50°C; at 60°C the enzyme displayed more than 70% of its original activity at 50°C, indicating that EstOF4 was moderately thermostable. The optimal activity was found to be around 50°C (Figure 6).

### Substrate specificity and kinetics

Different *p*-nitrophenyl esters were used as substrates to test the specificity of esterase EstOF4 and the results are given in Tables 1 and 2. The esterase showed hydrolysis activity towards short (pNPC2, 3.7 U/mg) and middle length p-nitrophenyl acylates (pNPC12, 0.15 U/mg). The highest activity was found toward *p*-nitrophenyl caproate (*p*NPC6, 10 U/mg). As the acylate chain is above *p*NPC12, no activity could be detected. The kinetic parameters were calculated by curve fitting to the Michaelis-Menten equation:
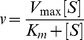
(1)

**Table 1:** Kinetic parameters for hydrolysis of the various p-nitrophenyl esters by purified EstOF4.

*p*- nitro phenyl esters	*K*_m_(µM)	*k*_cat_(min^−1^)	*k*_cat_/*K*_m_ (min^−1^·µM^−1^)
Acetate	37±6	124±4	3.4
Propionate	94±9	211±4	2.3
Butyrate	90±9	219±4	2.4
Caproate	60±6	329±6	5.5
Caprylate	102±3	105±2	1.0
Caprate	173±8	70±4	0.4
Laurate	42±5	6±1	0.1

All spectrometric assays were performed in the favourite active condition of the enzyme (50°C and pH 8.5).

where v is the conversion rate in µmol/min, V_max_ is the maximum velocity in µmol/min, *K*_m_ is Michaelis constant in µM and [S] is substrate concentration in µM. From the data of Table 1, the catalytic efficiency increased gradually with increasing chain length from *p*NC2 to *p*NC6, the highest catalytic efficiency *k*_cat_/*K*_m_ = 5.5 min^−1^·µM^−1^of the enzyme was found towards *p*-nitrophenyl caproate with the maximum *k*_cat_ of 329 min^−1^ and the lowest *K*_m_ of 60 µM. Further increase in the acylate chain to C8 resulted in the dramatic decrease of *k*_cat_ and increase of *K*_m_. Activity was also tested with triacylglycerol substrates using the pH-stat method. The highest activity was observed towards triacetin, at 1.3 U/mg (Table 2). The substrate profiles thus confirmed that EstOF4 was a carboxylesterase rather than a lipase, with the substrate preference to esters with short and medium chains.

**Table 2:** Substrate specificity of the esterase EstOF4 against p-nitrophenyl esters and glyceryl esters.

Substrate	U/mg
*p*-nitrophenyl acetate	3.7±0.3
*p*-nitrophenyl butyrate	6.6±0.5
*p*-nitrophenyl caproate	10±2
*p*-nitrophenyl caprylate	3.3±0.1
*p*-nitrophenyl caprate	1.7±0.2
*p*-nitrophenyl laurate	0.15±0.03
Triacetin	1.3±0.2
Tributyrin	0.5±0.05
Tricaproin	0.04±0.01

The assay was taken at the optimal condition (50°C and pH 8.5) with various substrates. Each value was averaged from three duplicates. The values shown represent the means ± standard deviations from the triplicate values measured.

### Inhibition studies

The effects of various compounds on the activity of esterase EstOF4 were shown in Table 3. Metal ions (Co^2+^, Ni^2+^, Fe^3+^) only showed minor inhibition to the activity of EstOF4 whilst Ca^2+^, Mg^2+^ managed 108% and 114% of the original activity. However, heavy metal ions such as Sn^2+^, Pb^2+^, Hg^2+^ and Cu^2+^ displayed a significant extent of inhibition. Organic solvent study showed that within 10% of variation dimethyl sulfoxide (DMSO) and acetonitrile did not affect EstOF4 activity. On the other hand, n-butanol, isopropanol and chloroform could cause further inhibition.

**Table 3:** Effects of different cations and reagents on the activities of EstOF4.

Metal ions (1 mM)	Relative activity	Organic solvent	Relative activity	Inhibitors	Relativeactivity
Blank	100				
Sn^2+^	36.4	10%DMSO	104.7	SDS 1 mM	6.5
Hg^2+^	10.7	10%Isopropanol	57.1	5 mM	2.3
Pb^2+^	11.7	10%Glycerol	109.5	EDTA 1 mM	91
Ag^1+^	11.6	10% n-butanol	6.6	5 mM	93.1
Cu^2+^	2.8	10%Acetone	49.3	DTT 1 mM	94.7
Mg^2+^	114.2	10%Chloroform	10.5	PMSF 1 mM	6.3
Ni^2+^	92.8	10%Acetonitrile	111	DEPC 1 mM	3.1
Fe^3+^	87.4	1%Tween20	37.5		
Ca^2+^	116	1%Tween60	58.9		
Co^2+^	114.1	1%Tween80	69.7		
Mn^2+^	107.6	1%TritonX100	49.6		

The assay was taken at the optimal condition (50°C and pH 8.5) with p-nitrophenyl caprate as substrate. The activity of EstOF4 in buffers without adding any ions was taken as 100%.

Non-ionic surfactants such as Tween 20, Tween 60, Tween 80 and Triton X100 didn't have any stimulating effect, but they instead reduced the activity of EstOF4 by at least some 40%. The serine specific inhibitor phenylmethanesulfonyl fluoride (PMSF) and histidine inhibitor diethylpyrocarbonate (DEPC) completely inhibited the enzyme, suggesting that serine and histidine were located in the esterase catalytic site and involved in the activity mechanism. Addition of 1 mM dithiothreitol (DTT) didn't influence enzymatic activity, indicating that the three cysteine amino acids in the protein didn't become involved in the catalytic activity of the enzyme. Chelating agent ethylenediaminetetraacetic acid (EDTA) didn't inhibit the activity of the esterase either. Anionic detergents such as SDS could dramatically inhibit the activity of EstOF4. For example, upon addition of 1 mM SDS, the activity dropped to less than 10% of its original value. This drastic activity loss might arise from the disruption of the dimeric structure or even the 3D conformational changes of the enzyme upon exposure to SDS. This feature is very different from lipases where surfactant binding often leads to the increase of activity due to the easy access to the active centre associated with the hydrophobic binding.

### Molecular structure modeling

**Figure 7:**
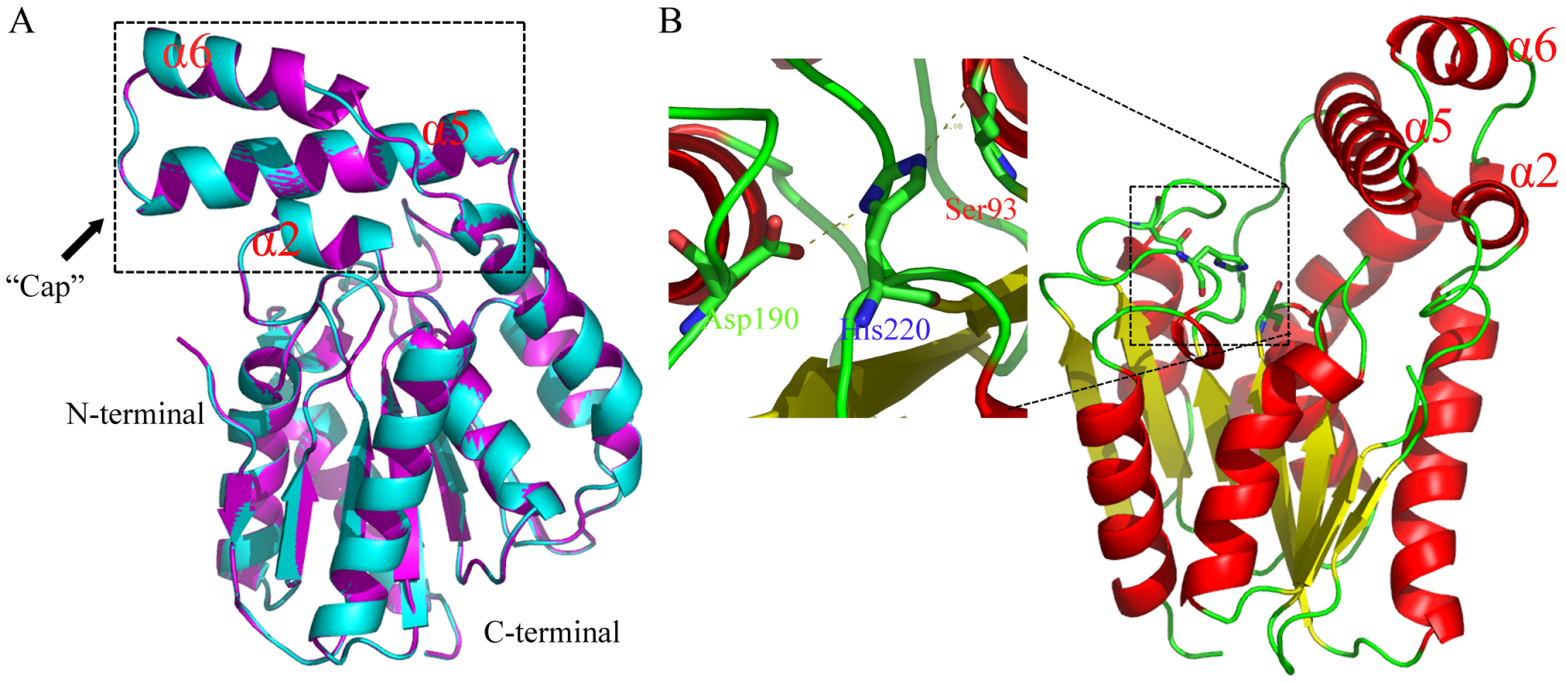
Homology model of EstOF4, produced by MODELLER 9.9 program based on the X-ray structure of esterase Est30 from Geobacillus stearothermophilus (GenBank Number AY186197,Protein Data Bank code 1TQH). Visualization of the tertiary structure was done by Pymol and represented in the form of ribbons. A: Superimposition of homology modelled structure of EstOF4 (magenta) onto the 3D structure of Est30 (green), the cap-like domain constructed by α2, α5 and α6 was enclosed in the dashed box. B: 3D model of EstOF4, with β-strands and α-helices shown in yellow and red, respectively, and the catalytic triad Ser93, Asp190 and His220 shown in sticks.

By searching RCSB PDB Protein Data Bank,the crystal structure of esterase Est30 from Geobacillus stearothermophilus was found as the most appropriate template for homology modeling, sharing a 71% amino acid sequence identity with EstOF4. Specifically, the residues in the catalytic triad and oxygen holes were the same for EstOF4 and Est30, Ser94, Asp193, His223, Phe25, Leu95 in Est30 corresponding to Ser93, Asp190, His220, Phe24, Leu94 in EstOF4. Both enzymes have similar functions and are the evolutional homology. A 3D structure model of the *B. pseudofirmus* esterase EstOF4 was constructed with software MODELLER 9.9 [21]. As validated by PROCHECK [22], the modeled structure has 94.7% residues in the most favored regions of Ramachandran plot, only 11 out of 247 amino acids in the additional and generously allowed regions (Figure S2). Such results indicate that the model is satisfactory. A superimposition of EstOF4 onto Est30 is shown in Figure 7A. As expected, the modeled EstOF4 possesses the structure with high similarity to Est30. As already indicated, the protein is composed of two domains, the catalytic α/β hydrolase fold domain (constructed by six α-helices surrounding seven β-sheets) and a cap-like domain (constructed with three helices α2, α5 and α6) (Figure 7B). Compared with its template, EstOF4 possesses a smaller cap domain due to the shorter α-helix domain.

As in Est30, EstOF4's catalytic triplet residues Ser93, Asp190 and His220 are located on the top of the catalytic domain and are close to the cap. In order to identify the catalytic triad, three mutant enzymes with Ser93Ala, Asp190Asn and His220Leu substitutions were respectively expressed and purified. None of these mutants showed lipolytic activity, confirming the importance of these residues on EstOF4's activity.

## Discussion

*B. pseudoufimus* OF4 is an aerobic gram-positive bacterium with optimal growth around pH 10.5 [14]. Part of its genome has recently been sequenced. One open reading frames encoding polypeptides showing high similarity to lipolytic hydrolases was found. After cloning the gene into recombinant expression system *E. coli* BL21, the lipolytic gene *estof4* was heterologously expressed in soluble form. Dimerization was critical for the enzyme to perform hydrolysis activity. Even with 1 mM SDS, the detergent could prohibit the formation of dimers which in turn diminished the enzymatic activity. Other detergents which could interrupt the dimeric structures of these enzymes were also effective at deteriorating bioactivity by causing unfolding of their native 3D structures essential for undertaking biocatalysis. Note that surfactants could also associate with the substrates which then became unrecognizable by the active site. This process could also result in the apparent loss of bioactivity, but requires experimental evidence to support.

Although EstOF4 was stable as dimer in native form when its concentration was low, it tended to aggregate when protein concentration increased.This aggregation tendency was also found in the esterases obtained from *Melanocarpus albomyce* [33] *Bacillus subtilis* (RRL1789) [34], because of the hydrophobic nature of these proteins. Enzymatic analysis of EstOF4 showed that the purified enzyme displayed metal independent hydrolysis activity towards short and middle chain *p*NP esters and triacylglycerol substrates with side chains shorter than C6, indicating that this enzyme was a true carboxylesterase instead of a lipase.

EstOF4 displayed moderate thermo-stability with the optimum temperature around 50°C, just as the esterases from *Geobacillus kaustophilus* [26], *Geobacillus stearothermophilus* [35], *Geobacillus thermoleovorans* [25] *and Bacillus coagulans* [28]. EstOF4 was active at neutral and alkaline pH with the optimal pH around 8.5. It was nevertheless not so alkaliphilic like extracellular lipases from alkaline bacteria such as *Burkholderia cepacia* ATCC 25416 [32] [36] and *Acinetobacter radioresistens* CMC-1 [37]. This difference probably arises from the fact that EstOF4 is an intracellular enzyme active in the cytoplasm of alkaliphilic *bacillus pseudofirmus* OF4. The cytoplasm pH of alkaliphilic microorganisms is at relatively lower alkaline level than the outside environment [38]. This observation is in agreement with the fact that no signal peptide was found when the protein sequence was analyzed.

Two typical esterases, Est30 [11] and HTA426 [26] from the newly defined bacterial family (Family XIII), were aligned with the sequence of EstOF4. From the sequence alignment we found a conserved pentapeptide GLSLG in these proteins and the putative catalytic triad (Ser93, Asp222 and His240) (Figure 2). These structural features were also confirmed by amino acid mutagenesis. With the available 3D molecular structure of esterase Est30, a modeled structure of EstOF4 is constructed. The EstOF4 3D structural model showed a typical α/β hydrolase fold constructed with a central 7 β-sheet barrel surrounded by 6 α-helices [12]. Most obviously, EstOF4 has a molecular structure with a small cap domain characteristic of Est30. The fact that EstOF4 is active under most solution conditions suggests that this cap domain doesn't prohibit the active site like the “lid domain” in many lipases. Its catalytic residue Ser93 must be accessible to the substrate molecules just as in esterase Est30.

Bacterial lipases/esterases make important contributions to the universal lipolytic hydrolases in the environment. Although these enzymes can have a lot of differences in primary sequence, they share similar molecular features and perform similar catalytic activities towards esters, implying that they are originally related. Since Arpigny and Jaeger first grouped all these ester hydrolases into 8 families some 10 years ago [7], there are now 14 families, showing fast advances in discovering bacterial lipolytic hydrolases (Figure 1). At the time of creating Family XIII, Est30 was the typical thermostable esterase representing the newest family by possessing the unique structural topology with the 6α/7β tertiary hydrolyase folds, making it highly interesting for further investigation [11], [12]. From a careful comparison of the hitherto discovered lipolytic families, we found that EstOF4 and the other 5 homologous enzymes (AAX86643, AAT57903, AAT65181, ABB90597 and BAD77330) can be grouped in the same cluster as Est30 (1TQH) under the phylogenetic tree (Figure 1). It is obvious from the sequence alignment that this family shares a typical pentapeptide motif (GLSLG) for all the esterases, different from the conserved pentapeptide GXSMG in Family V and another conserved pentapeptide GFSQG in Family VI [7], even though all these enzymes shared most homologous identities within these families. Although previous work has analyzed the enzymatic properties of the other six homologous esterases, their phylogenetic status in the bacterial lipolytic enzymes hasn't been properly analyzed. From the highly conserved sequence identity it is reasonable to group all these enzymes into the same family of Est30 to form Family XIII.

Based on their enzymatic properties (Table 4), we can conclude that enzymes in Family XIII have specific hydrolysis preference towards short chain ester substrates and that they mostly show their optimal performance from neutral to alkali pH. Even though their enzymatic properties are similar their thermal adaptive properties vary. The optimal temperature is 79°C for Est30 from *Geobacillus stearothermophilus* [11] and 65°C for EstA from *geobacillus thermoleovorans* [25]. Esterase EstOF4 from this study could just rank as the moderate thermostable member of this family with the optimal temperature of 50°C. It is however interesting to note that the most psychrophilic one from *Bacillus cereus* has the optimal temperature of 33°C [27]. From the diverse thermostability of all these highly sequence identical enzymes we could see that they adopt in high temperature environment by changing only a few parts of their amino acids. This is a crucial clue to guide enzymatic modification.

**Table 4:** Biochemical properties of homologous esterases in bacterial lipolytic Family XIII from various organisms.

Origin	Protein Name	GenBank Number	OptimumpH &Temp(°C)		Optimum Subst(pNP)	Properties	Ref
*Geobacillus stearothermophilus*	Est30	AY186197(1TQH)	9	79	C6	thermostable	[11]
*Geobacillus thermoleovorans*	EstA	ABB90597	9.5	65	C2	thermostable	[21]
*Geobacillus kaustophilus*	HTA426	BAD77330	8.0	60	C2	thermostable	[22]
*Bacillus pseudofirmus*	EstOF4	YP003426005	8.5	50	C6	Moderate thermostable	This study
*Bacillus coagulans*	Carboest	AAT57903	8	50	C4	Moderate thermostable	[24]
*Bacillus sp*	EstB2	AAT65181	7	35	C4	stable in high salt	[20]
*Bacillus cereus*	Est	AAX86643	9	33	C4	enantio-selectivity	[23]

## Supporting Information

Figure S1 **Identification of the native form of EstOF4 by gel filtration chromatography with a Superdex-200 column.** The elution volumes of standard proteins are cytochrome c 17.3 ml, carbonic anhydrase 16.0 ml, albumin bovine13.5 ml, alcohol dehydrogenase 12.5 ml andβ-amylase 11.4 ml. The elution volume of esterase EstOF4 is 13.8 ml.
(TIF)

Figure S2 Click here for additional data file. **Ramachandran plot for the theoretical model of EstOF4.** Residues in most favoured regions [A,B,L] 196 94.7%;Residues in additional allowed regions [a,b,l,p] 10 4.8% Residues in generously allowed regions [∼a,∼b,∼l,∼p] 1 0.5%.
(TIF)

File S1 Click here for additional data file. (DOC)
