# Supplementary figures and images for: A Novel Alkaliphilic Bacillus Esterase Belongs to the 13th Bacterial Lipolytic Enzyme Family
Source: PLoS One. 2013 Apr 5;8(4):e60645. doi: 10.1371/journal.pone.0060645 (PMC3618048; doi:10.1371/journal.pone.0060645)

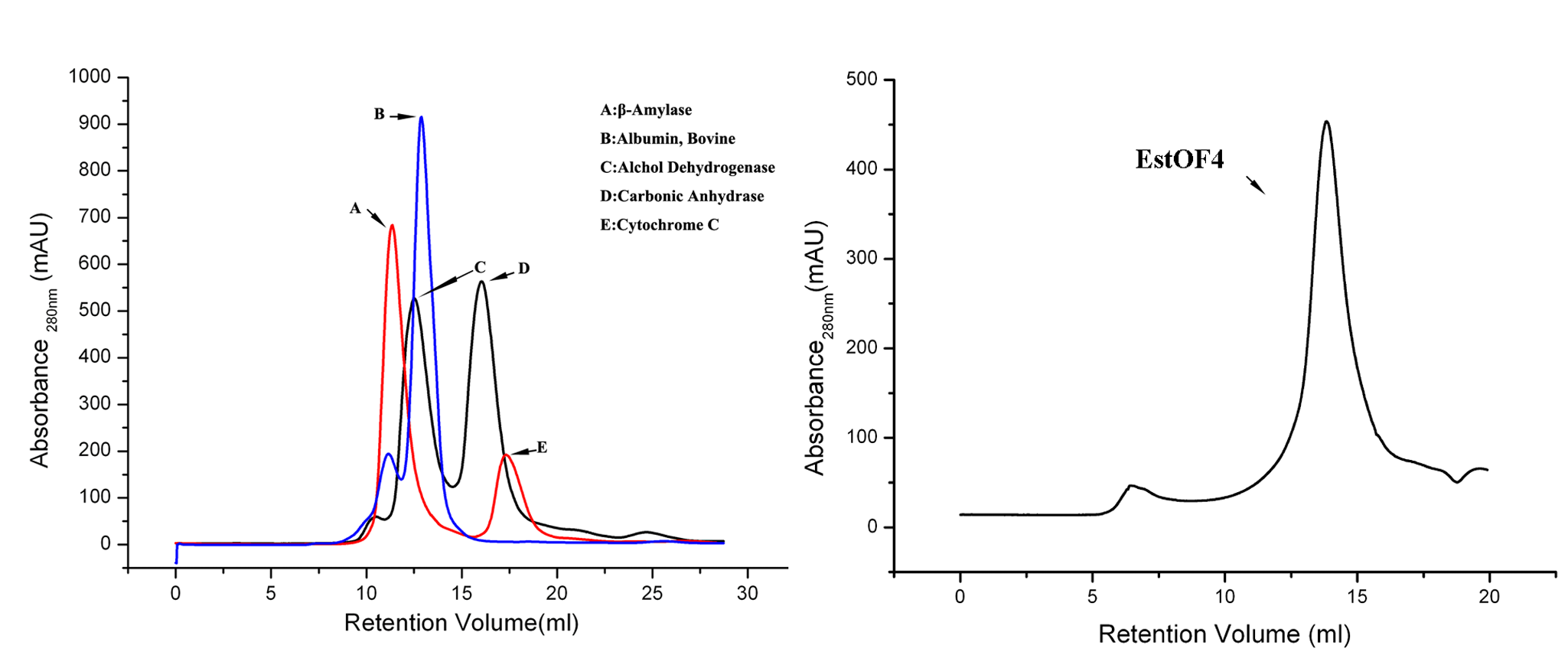

Supplement: Figure S1 — Identification of the native form of EstOF4 by gel filtration chromatography with a Superdex-200 column. The elution volumes of standard proteins are cytochrome c 17.3 ml, carbonic anhydrase 16.0 ml, albumin bovine13.5 ml, alcohol dehydrogenase 12.5 ml andβ-amylase 11.4 ml. The elution volume of esterase EstOF4 is 13.8 ml. (TIF) [file pone.0060645.s001.tif]

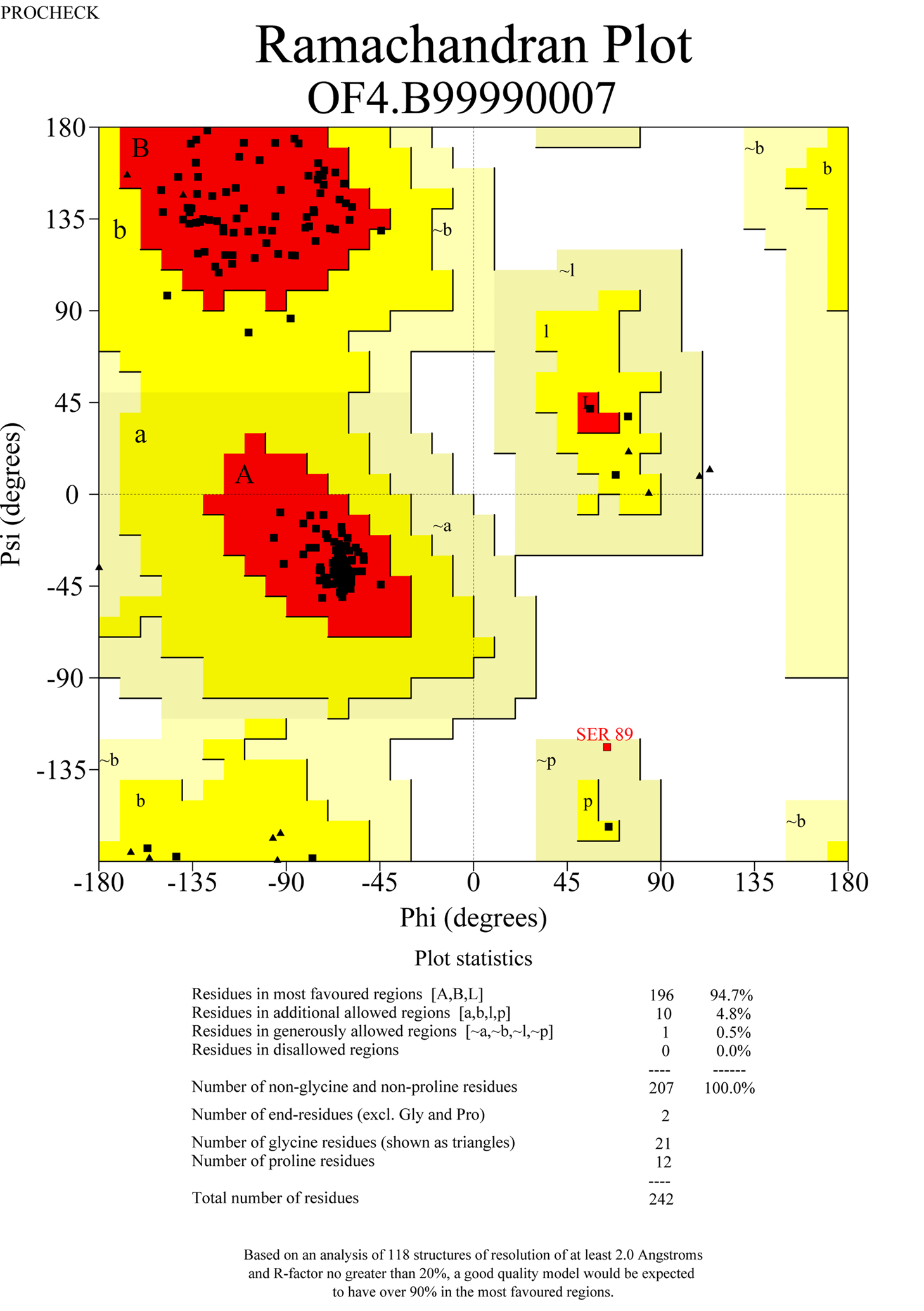

Supplement: Figure S2 [file pone.0060645.s002.tif]
