# Supplementary material for: A Novel Alkaliphilic Bacillus Esterase Belongs to the 13th Bacterial Lipolytic Enzyme Family
Source: PLoS One. 2013 Apr 5;8(4):e60645. doi: 10.1371/journal.pone.0060645 (PMC3618048; doi:10.1371/journal.pone.0060645)
Supplement: File S1 [file pone.0060645.s003.doc]

Supplementary Information (PONE-D-12-40057)

# A novel alkaliphilic Bacillus esterase belongs to the 13^th^ bacterial lipolytic enzyme family

Lang Rao^1,2^, Yanfen Xue^1^, Yingying Zheng ^1,2^, Jian R. Lu^3^﹡and Yanhe Ma^1^﹡

1. State Key Laboratory of Microbial Resources, Institute of Microbiology, Chinese Academy of Sciences, Beijing 100101, China
2. The Graduate School, Chinese Academy of Sciences, Beijing 100049, China
3. Biological Physics Laboratory, School of Physics and Astronomy, the University of Manchester, Manchester M13 9PL, United Kingdom

*Figure S1: Identification of the native form of EstOF4 by gel filtration chromatography with a Superdex-200 column. The elution volumes of standard proteins are cytochrome c 17.3ml, carbonic anhydrase 16.0ml, albumin bovine13.5ml, alcohol dehydrogenase 12.5ml andβ-amylase 11.4ml. The elution volume of esterase EstOF4 is 13.8ml.*


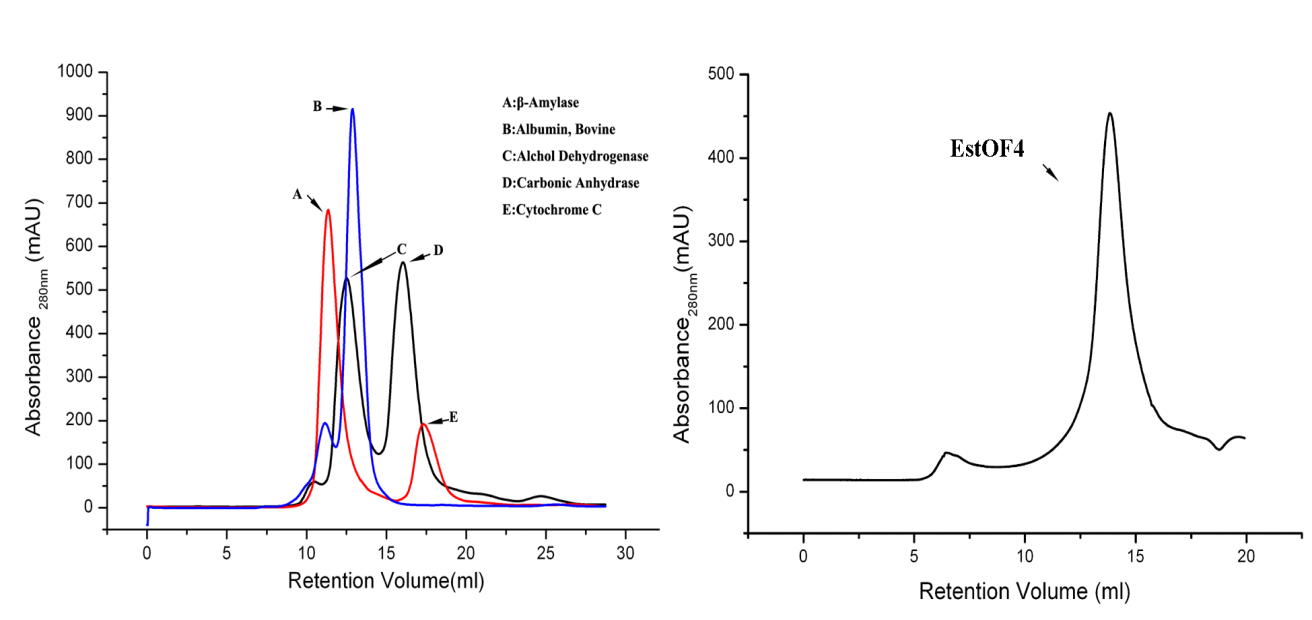


*Figure S2 Ramachandran plot for the theoretical model of EstOF4. Residues in most favoured regions [A,B,L]* *196 94.7%;Residues in additional allowed regions [a,b,l,p] 10 4.8%*

*Residues in generously allowed regions [~a,~b,~l,~p] 1 0.5%*

*Table S1 Purification of the recombinant enzyme EstOF4*

| Step | Total protein (mg） | Total activity (U) | Specific activity (U/mg) | Yield (%) | Purification  (fold) |
| --- | --- | --- | --- | --- | --- |
| Crude extract | 1352 | 1152 | 0.85 | 100 | 1 |
| Ni column | 84 | 571 | 6.8 | 50 | 8 |
| Gel filtration | 22 | 230 | 11 | 20 | 13 |

Activity was taken using pNC6 as substrate at 50 *^o^C*.
